# Macrophage immunometabolism in osteoporosis: an emerging regulatory axis linking metabolic–polarization coupling to therapeutic targets

**DOI:** 10.3389/fimmu.2026.1953599

**Published:** 2026-09-28

**Authors:** Lian Weng, Qianli Yuan, Taizhou Mo, Taiming Yuan, Zhongwei Xiong, Liao Ding

**Affiliations:** 1 The Second Department of Orthopedics, Luzhou Longmatan District People's Hospital, Luzhou, Sichuan, China; 2 Department of Orthopedics, Affiliated Hospital of Southwest Medical University, Luzhou, Sichuan, China; 3 Department of Orthopedics, The First Affiliated Hospital of Chongqing Medical University, Chongqing, China; 4 Department of Orthopedics, The Affiliated Traditional Chinese Medicine Hospital of Southwest Medical University, Luzhou, Sichuan, China

**Keywords:** bone remodeling, histone lactylation, immunometabolism, macrophage polarization, metabolic reprogramming, osteoporosis

## Abstract

Osteoporosis has long been understood, and treated, as an imbalance between osteoblastic bone formation and osteoclastic resorption. Yet many patients continue to lose bone, pointing to upstream regulators of the remodeling unit that remain poorly defined. Macrophages are developmentally continuous with osteoclasts and functionally coupled to bone-forming cells, and are now recognized as central immune regulators of skeletal homeostasis. What sets their polarization state, however, has been comparatively neglected in bone. This review adopts metabolic–polarization coupling as a unifying framework for macrophage function in osteoporosis. We argue that the metabolic program a macrophage selects acts as an upstream regulator that biases its phenotype and, in turn, the balance of bone resorption and formation, and we examine how this switch is reinforced epigenetically, amplified by comorbidity, and rendered therapeutically actionable. Aerobic glycolysis via the HIF-1α axis, versus oxidative phosphorylation and fatty-acid oxidation, sets M1 versus M2 phenotype and thereby the balance of resorption and osteogenic–vascular support. Lactate-derived post-translational modifications, notably histone lactylation, make this metabolic state durable at the epigenetic level, while metabolic comorbidities, including ageing, diabetes and obesity, amplify the axis to accelerate bone loss. We emphasize at the outset that direct intra-osseous evidence for this axis remains scarce: the skeletal phenotypes conferred by macrophage-specific deletion of HIF-1α or CPT1A are unreported, histone lactylation has yet to be quantified in bone-resident macrophages, and much of the mechanistic support stems from non-skeletal tissues, inflammatory bone-loss, or periodontal models rather than from primary postmenopausal or age-related osteoporosis. Accordingly, throughout this review we explicitly distinguish evidence established within bone from evidence merely extrapolated to bone. Repositioning macrophage metabolism from a passive correlate to an upstream causal node yields an emerging regulatory axis yields a testable sequence, metabolic node to polarization to remodeling imbalance to bone loss, that identifies metabolic enzymes such as HIF-1α and CPT1A as candidate druggable targets and polarization and lactylation signatures as candidate readouts for stratification. This perspective reframes osteoporosis therapy from suppressing mature osteoclasts toward reprogramming macrophage metabolism itself.

## Introduction

Osteoporosis is among the most prevalent metabolic skeletal disorders of the ageing population. Progressive loss of bone mass and microarchitectural deterioration culminates in fragility fractures, disability, and excess mortality (1). For decades, the prevailing framework explained this disease almost entirely as a disturbed balance between osteoblast-mediated bone formation and osteoclast-mediated bone resorption (2). Therapeutics built on this framework, antiresorptives and anabolic agents, have reduced fracture risk. Yet many patients continue to lose bone or fail to respond. This implies that upstream regulators of the remodeling unit remain incompletely understood.

A major conceptual shift has come from the recognition that bone and the immune system are deeply intertwined, a field now termed osteoimmunology (3). Within this framework, macrophages have emerged as pivotal immune regulators of skeletal homeostasis. Bone-resident macrophages (osteomacs), together with osteoclast precursors of the monocyte–macrophage lineage, couple physically and functionally to osteoblasts and osteoclasts. They thereby coordinate both physiological remodeling and pathological bone loss (4, 5). Their functional identity is commonly framed along the M1 (pro-inflammatory) versus M2 (reparative) polarization axis. This balance shapes the local cytokine environment that governs osteoclastogenesis and osteogenic support (6).

Despite growing attention to macrophage polarization in bone, a fundamental question has remained comparatively neglected in the skeletal field: what determines the polarization state in the first place? In other tissues, the answer is now firmly established. The metabolic program of a macrophage (its reliance on glycolysis versus oxidative phosphorylation, its handling of lipids and amino acids) acts as an upstream switch that dictates phenotype and effector function (7). This principle, immunometabolism, reframes polarization not as a fixed endpoint but as the readout of an underlying metabolic state. Its systematic application to osteoporosis, however, is only beginning to take shape.

Recent evidence indicates that this metabolic layer is directly relevant to the bone microenvironment. Metabolic reprogramming of macrophages has been proposed as a new perspective for bone healing and disease (8). In parallel, lactate-derived histone lactylation, characterized as a metabolite-driven epigenetic mark linking TLR-driven glycolysis to chromatin remodeling in macrophages (9), is now implicated in bone health and disease. It acts as a mechanistic bridge between a cell’s metabolic status and its transcriptional program (10). These observations, still fragmentary, converge on a coherent but underexplored axis: macrophage metabolic reprogramming operates upstream of polarization to shape skeletal fate.

Existing reviews have documented the association between macrophage polarization and osteoporosis, but they largely stop at the downstream “M1/M2-to-bone” relationship (11, 12). What has been missing is an integrative account that places upstream metabolic reprogramming, and its epigenetic outputs, at the organizing center of this relationship. In this review, we adopt “metabolic–polarization coupling” as a unifying framework and trace a single mechanistic chain across the osteoporotic bone microenvironment: metabolic reprogramming → macrophage polarization → imbalance of resorption and formation → bone loss (**Figure 1**). We further examine how metabolic comorbidities such as diabetes, obesity, and ageing feed into this axis, and we translate the mechanistic picture into a landscape of druggable immunometabolic targets. This review recasts macrophage metabolism from a passive correlate of bone remodeling into a candidate upstream regulator. The claim is framed with due caution: causal evidence generated within osteoporotic bone is sparse next to that built up in lung, tumor, cardiovascular and adipose tissue, and selective deletion of a defining metabolic node in the macrophage lineage has yet to be linked to any bone phenotype. We thus present this axis as an emerging and testable framework rather than an established determinant, and throughout we keep distinct what has been shown in bone and what is only inferred from other tissues. On that basis, it offers a mechanistically grounded and clinically oriented perspective on osteoporosis. The aim is to complement the existing polarization-centric literature, not to restate it.

**Figure 1:**
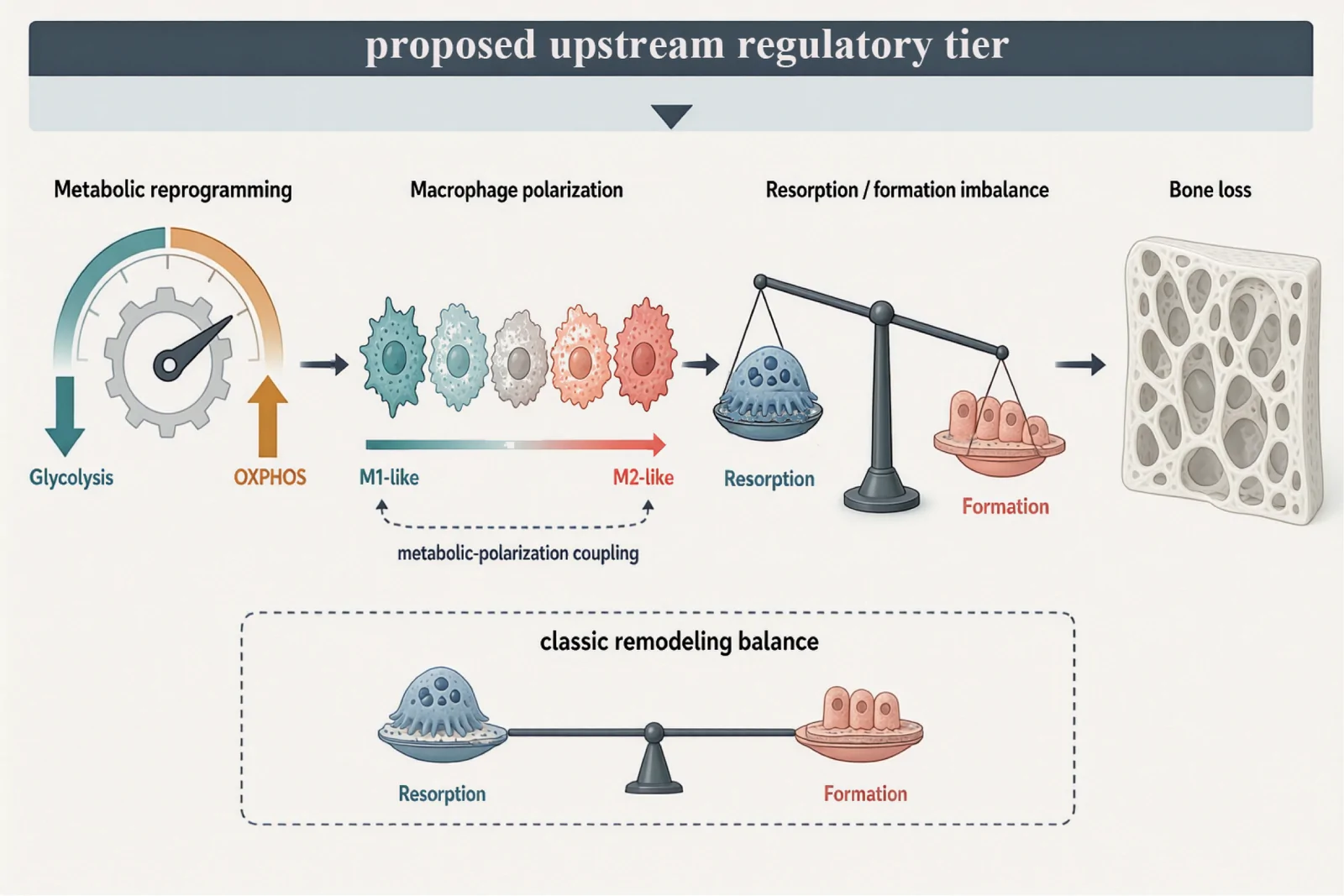
Macrophage immunometabolism as a candidate upstream regulatory axis of bone remodeling: the metabolic–polarization coupling framework. This conceptual overview presents macrophage metabolic reprogramming as a candidate upstream regulator, rather than a downstream correlate, of bone remodeling; the framework is proposed as a testable axis and not as an established causal chain. A left-to-right causal chain links metabolic reprogramming (glycolysis–OXPHOS balance) to macrophage polarization along the M1–M2 continuum, to an imbalance between osteoclastic resorption (RANKL↑) and osteoblastic formation, and ultimately to trabecular bone loss. A thick arrow spanning the chain denotes metabolic–polarization coupling; the top bar marks the proposed upstream regulatory tier, and the dashed box below shows the classical formation/resorption model for old-versus-new comparison. M1 and M2 are depicted as metabolic read-outs rather than fixed endpoints. This framework organizes the mechanistic chain developed throughout the Review.

## Literature search strategy

This review draws on a structured, iterative search of PubMed/MEDLINE, extended by forward and backward citation tracking of every retrieved article and by hand-searching the reference lists of recent reviews of osteoimmunology, immunometabolism and lactylation. Publications published between January 1990 and June 2026 were considered; no lower date limit was applied so that the landmark descriptive studies that defined the RANKL–OPG axis, osteoclast ontogeny and the M1/M2 nomenclature could be retained. Queries were built across four concept blocks and combined with Boolean operators, using controlled vocabulary where available: (i) cells and tissues (‘macrophage’, ‘monocyte’, ‘osteomac’, ‘osteal macrophage’, ‘osteoclast precursor’, ‘bone marrow macrophage’); (ii) metabolism (‘immunometabolism’, ‘metabolic reprogramming’, ‘glycolysis’, ‘oxidative phosphorylation’, ‘fatty-acid oxidation’, ‘HIF-1α’, ‘CPT1A’, ‘mTOR’, ‘AMPK’, ‘NRF2’, ‘SIRT1’, ‘glutaminolysis’, ‘itaconate’, ‘succinate’); (iii) epigenetics (‘lactylation’, ‘histone lactylation’, ‘H3K18la’, ‘succinylation’, ‘acetylation’, ‘post-translational modification’); and (iv) the skeleton (‘osteoporosis’, ‘bone loss’, ‘bone remodeling’, ‘osteoclastogenesis’, ‘bone mineral density’, ‘inflammatory bone loss’, ‘periodontitis’).

Records were screened by title and abstract and then in full text. Studies were included if they were peer-reviewed primary reports or authoritative syntheses published in English that (a) defined a specific metabolic or metabolite-derived epigenetic node in macrophages or the macrophage–osteoclast lineage, (b) reported a skeletal, osteoclastogenic or osteogenic readout, or (c) established a mechanistic principle in a non-skeletal tissue that is explicitly invoked in this review as an extrapolation. Studies were excluded if they were conference abstracts, non-peer-reviewed preprints, case reports, or non-English publications, if they reported polarization phenotype without any metabolic or epigenetic measurement, or if the metabolic claim could not be traced to primary data. Because the field is young and the skeletal evidence base is small, no formal risk-of-bias scoring or meta-analytic synthesis was attempted; this is a narrative, mechanism-oriented review and not a systematic review, and it is not registered as such. To keep the strength of each claim visible, every included study was additionally classified by model class, as primary osteoporosis (ovariectomy, ageing or glucocorticoid-induced), inflammatory or periodontal bone loss, or non-skeletal tissue, and that classification is reported in **Table 1** and used throughout the text to separate bone-established from extrapolated evidence.

**Table 1:** Landmark studies linking macrophage metabolic reprogramming to skeletal phenotypes.

Model / system	Model class	Key metabolic node	Macrophage / osteoclast readout	Skeletal phenotype	Evidence level	Study (first author, year) [ref]
Rodent + cell (preclinical)	Skeletal (rodent + cell); primary-osteoporosis status not specified in source	HIF-1α–driven glycolysis	Macrophage metabolic reprogramming	Osteoblast ferroptosis and bone loss (via IL-6–STAT3)	Bone-direct, *in vivo* + *in vitro*	Gu et al., 2026 (32)
Murine periodontitis (*in vivo*)	Inflammatory bone loss (periodontitis)	HIF-1α stabilization (DMOG)	Macrophage polarization	↓ Alveolar bone resorption	Bone-direct, *in vivo* (inflammatory model)	Chen et al., 2021 (26)
Osteoclast lineage (preclinical)	Inflammatory bone resorption (osteoclast lineage)	LSD1–HIF-1α–E2F1	Osteoclast differentiation	Inflammatory bone resorption	Bone-direct	Doi et al., 2022 (36)
Osteoporosis model (preclinical)	Primary osteoporosis	FAO→glycolysis switch (PRMT6)	Osteoclast differentiation	Osteoporotic bone loss	Bone-direct	Chu et al., 2024 (37)
Mouse (*in vivo*)	Skeletal (osteoclast lineage); not an osteoporosis model as reported	Glutaminolysis (nucleotide / amino-acid supply)	Osteoclast differentiation	Osteoclastogenesis regulated	Bone-direct, *in vivo*	Hu et al., 2024 (38)
Endothelium → BMSC (preclinical)	Primary osteoporosis (non-macrophage lineage: endothelium to BMSC)	Lactate → histone lactylation	Endothelial lactate → BMSC lactylation	Attenuated osteoporosis	Bone-direct	Wu et al., 2023 (51)
Osteoblast (*in vitro*)	Non-osteoporosis *in vitro* (osteoblast lineage)	Lactate → H3 lactylation (p300)	Osteoblast histone lactylation	↑ Osteoblast differentiation	Bone-direct, *in vitro*	Minami et al., 2023 (52)
Inflammatory bone loss (*in vivo*)	Inflammatory bone loss	Mitophagy (STAT3)	Macrophage NLRP3 inflammasome activation	Inflammatory bone loss	Bone-direct, *in vivo*	Zhu et al., 2023 (62)
Systemic inflammatory bone loss (*in vivo*)	Inflammatory bone loss (systemic)	Mitophagy (APJ–BNIP3–PINK1–PARKIN)	Macrophage mitochondrial turnover	↓ Systemic inflammatory bone loss	Bone-direct, *in vivo*	Wang et al., 2025 (63)
Preclinical (*in vivo*)	Primary osteoporosis	Proteostasis: REGγ–TRAF6 degradation	Osteoclast differentiation	Prevents osteoporotic bone loss	Bone-direct, *in vivo*	Du et al., 2024 (65)
Preclinical (*in vivo*)	Primary osteoporosis	Lipid-raft organization (stomatin)	Osteoclastogenic threshold	Ameliorated osteoporosis	Bone-direct, *in vivo*	Tao et al., 2025 (66)
Bone marrow (preclinical)	Skeletal (marrow / vascular niche); not an osteoporosis model as reported	Myeloid differentiation control (RKIP)	Osteoclastogenesis + type H vessel formation	Angiogenic–osteogenic niche / remodeling	Bone-direct	Zheng et al., 2025 (67)
Preclinical (*in vivo*)	Primary osteoporosis (skeletal ageing)	Immunosenescence (grancalcin release)	Senescent immune cells	Skeletal aging / bone loss	Bone-direct, *in vivo*	Li et al., 2021 (78)
Diabetes / periodontal (*in vivo*)	Inflammatory bone loss (diabetic periodontitis)	ROS overproduction (high-glucose-driven	Macrophage M1 polarization (M2 suppression)	Diabetic periodontal bone loss	Bone-direct (comorbidity)	Zhang et al., 2021 (83)
Age-related osteoporosis (preclinical)	Primary osteoporosis (ageing)	Macrophage senescence (β-catenin/FOXO1/REDD1)	Macrophage senescence restraint	Protection against age-related osteoporosis	Bone-direct	Cheng et al., 2024 (80)
Human and mouse bone-lining tissue (*in vitro* + *in vivo*)	Non-osteoporosis skeletal (bone-lining tissue / healing)	Oxidative, trophic resident-macrophage programme (metabolism not directly profiled)	Osteomac identity and requirement for osteoblast mineralization	Supports bone formation; depletion impairs mineralization and intramembranous healing	Bone-direct; metabolic characterization absent	Chang et al., 2008 (95); Alexander et al., 2011 (96)
Osteoclast lineage (*in vitro* + *in vivo*)	Skeletal (osteoclast lineage); not an osteoporosis model as reported	ATP-citrate lyase; nucleocytosolic acetyl-CoA supply	Histone acetylation in osteoclast precursors	ACLY inhibition suppresses osteoclast differentiation and function	Bone-direct; only metabolite-derived modification with a causal skeletal cell-fate link	Guo et al., 2021 (103)
Murine skeletal development (*in vivo*, genetic)	Non-osteoporosis skeletal (development)	HIF-α pathway (osteoblast lineage)	Osteoblast-lineage HIF-α; vascular coupling	Increased bone mass via angiogenic–osteogenic coupling; illustrates the dual, cell-type-opposed role of HIF	Bone-direct, *in vivo* genetic; counterweight to systemic HIF inhibition	Wang et al., 2007 (104)

(Evidence caveat): much of the *in vivo* causal evidence derives from inflammatory or systemic bone-loss models rather than primary postmenopausal / age-related osteoporosis; extrapolation to the commonest clinical forms remains to be tested. Reference numbers in ( ) correspond to the numbered reference list.

## Macrophages in the bone immune microenvironment

Which macrophages inhabit bone, where they reside, and how their functional states map onto skeletal homeostasis: these questions ground everything that follows about their metabolism. The bone surface and marrow harbor a heterogeneous population of mononuclear phagocytes. Their identities are defined as much by anatomical location as by phenotype. A distinct resident population, the bone-lining or “osteomac,” sits in intimate contact with osteoblasts along active remodeling surfaces, contributing to bone formation and repair, not resorption (5). Osteomacs were first characterized as F4/80+ cells distributed throughout the bone-lining tissue of humans and mice, where they are required for osteoblast-driven mineralization both in culture and *in vivo* (95). Their selective ablation disrupts intramembranous bone healing, which has led to their being viewed as a functionally indispensable, pro-osteogenic population rather than as a pool of cells destined for resorption (96). In the metabolic argument developed below, two properties set them apart from osteoclast precursors. The first is developmental in origin. Osteomacs behave as self-sustaining, tissue-resident cells of the kind now recognized in many organs, whereas osteoclast precursors are recruited monocytes that differentiate only in response to RANKL (91). The second concerns the nature of their activity. Osteomacs perform oxidative, trophic work rather than resorptive work, and on first principles they would therefore be expected to metabolize through oxidative phosphorylation and fatty-acid oxidation, in line with reparative macrophages; osteoclast commitment, by contrast, is accompanied by a well-documented switch toward glycolysis and glutaminolysis (35, 37, 38). We emphasize that this expectation remains untested. To our knowledge, no study has profiled the metabolism of purified osteomacs, and no published comparison yet exists of substrate utilization, mitochondrial content or extracellular acidification rate between osteomacs and osteoclast precursors derived from the same bone. Characterizing the metabolic phenotype of the osteomac compartment therefore stands out among the gaps this Review identifies as immediately addressable. These tissue-resident cells are functionally separable from the recruited, marrow-derived phagocytes that supply osteoclast precursors. Across the remodeling cycle, macrophage subsets in bone are now understood to occupy a spectrum of roles (4). The marrow compartment complicates the picture further. Adipocyte-biased progenitors there can promote osteoclastogenesis, showing that the local cellular environment, not the phagocyte alone, shapes resorptive output (13). This anatomical and functional heterogeneity is the essential backdrop to the immunometabolic arguments of later sections.

The tightest and most consequential relationship in this microenvironment is the developmental continuity between macrophages and osteoclasts. Osteoclasts are the sole cells capable of resorbing mineralized bone. They arise from the monocyte–macrophage lineage, and their differentiation and resorptive activity have long anchored the classical model of bone turnover (14). The molecular basis of this control was established by the discovery of the receptor activator of nuclear factor-κB ligand (RANKL)–osteoprotegerin (OPG) axis. OPG was identified as a secreted decoy receptor that restrains bone resorption and preserves bone density (15). Its ligand RANKL was defined as the cytokine that drives osteoclast differentiation and activation (16). Genetic evidence then confirmed RANKL/OPGL as a master regulator of osteoclastogenesis *in vivo* (17). Subsequent work refined this view: it established osteoclasts as dynamic, regulable effectors, not terminal end-cells, and set the benchmark for how resorption is controlled at the cellular level (2). A more recent and striking revision is the identification of the “osteomorph.” In this state, osteoclasts undergo fission and recycling instead of simple apoptosis, redistributing their nuclei and reforming resorptive cells over time (18). This recycling behavior recasts the macrophage–osteoclast axis as a renewable, reversible system, with clear implications for how metabolic and pharmacological interventions might act. Much of the mechanistic dissection of these programs has relied on tractable models such as RAW264.7 monocytic cells. These remain a standard tool for interrogating osteoclast formation (19).

If lineage continuity defines who becomes an osteoclast, polarization defines how the non-resorptive macrophage pool influences the balance between bone loss and bone gain. Macrophages in bone adopt a continuum of activation states, conventionally simplified as pro-inflammatory (M1) and reparative (M2) poles. The relative dominance of these states is now recognized as a central lever of bone-immune regulation (20). An M1-skewed environment favors an inflammatory setting that supports osteoclastogenesis and resorption. M2-associated programs, by contrast, are linked to the resolution of inflammation and to trophic support of bone-forming cells. The osteoimmunological literature thus frames macrophage polarization as a direct determinant of bone homeostasis, not a bystander of it (6). This polarization framework is more than descriptive. It marks the interface at which systemic and local signals are translated into skeletal outcomes. On these grounds, the interaction between bone and immune cells is invoked to explain postmenopausal and inflammatory bone loss (3). Establishing this M1/M2 axis here, without yet dissecting its metabolic control, provides the functional target that the remainder of this review addresses.

The M1/M2 shorthand employed earlier — and kept throughout this Review only for economy of expression — calls for a qualifying remark, since read at face value it distorts macrophage biology. Long-standing consensus guidance on nomenclature warns that M1 and M2 refer to idealized *in vitro* states brought on by individual stimuli, rather than to stable cell types that tissue can yield; activation, it is recommended, should be characterized by the applied stimulus and marker panel and not by a two-state label (89). Network-level transcriptomic profiling of human macrophages challenged with a diverse stimulus set has uncovered a continuum of activation programs reaching well past the canonical poles, with several programs having no representation within the binary scheme (90). Macrophages resident in tissue introduce a further axis that the M1/M2 frame is unequipped to handle: phenotypic heterogeneity *in situ* is governed principally by ontogeny and niche imprinting, with acute polarizing cues playing a subordinate part (91). The bone setting sharpens the issue. Single-cell dissection of the marrow microenvironment (92) and stromal taxonomies of the marrow (93) reveal a myeloid compartment carved into several transcriptional states whose limits do not map onto M1/M2, while a recent single-cell atlas of regenerating bone positions itself as moving “beyond polarization,” assigning macrophage subsets instead through neuro-immune and matrix-engagement programs (94). The clearest case is the osteomac. Although it supports formation and is therefore often tagged as “M2-like,” it is in fact a resident lineage of distinct ontogeny with, as noted, an uncharacterized metabolism; treating it as an interleukin-4-driven M2 macrophage is consequently a projection, not a measurement.

Our usage is therefore stated openly. Whenever this Review refers to “M1-skewed” or “M2-associated” states, it marks a position on a continuous gradient between inflammatory and reparative tendencies, inferred in most of the cited literature from a limited set of markers — typically iNOS, TNF-α and IL-1β on one side and CD206, ARG1 and IL-10 on the other — together with a metabolic readout; the phrase should be read as shorthand for that experimentally measured bias and not as a claim of discrete cellular identity. The two-state simplification is retained in Sections 3, 5 and 7 because the primary studies on which those arguments rest themselves rely on two-pole phenotyping: the glycolysis- and HIF-1α-centered reports (24–26, 32), the mitophagy–inflammasome and vesicle studies (62, 63, 71, 86) and the comorbidity studies (81, 83) all categorize macrophages dichotomously, and describing them in other terms would overstate their resolution. Finer classifications are used only where the source data warrant them: the single-cell studies noted earlier resolve marrow myeloid states without invoking M1/M2 (92–94); the osteomac literature defines a resident population by anatomical location and surface phenotype rather than by polarization (5, 95, 96); and the osteomorph work identifies a recycling osteoclast state that no polarization label captures (18). This has two implications for the argument developed here. First, the metabolic profile of bone-resident osteomacs cannot be presumed to coincide with that of a canonical M2 macrophage, and the possibility that formation-supporting resident macrophages are sustained by a bone-specific metabolic program remains an open question. Second, because polarization is graded, the therapeutic aim is better formulated as shifting a population distribution than as converting one cell type into another — a point we revisit in Section 7.

The clinical relevance of this framework becomes evident when polarization balance is perturbed. In estrogen-deficient and chronically inflammatory states, the macrophage compartment adopts an activation profile that tips remodeling toward resorption. This immunological imbalance is increasingly regarded as a driver of osteoporotic bone loss, not a secondary consequence of it (3). Under overt pathological conditions the resorptive arm is amplified, with osteoclasts acting as the proximate effectors of the resulting bone destruction (21). Such disease-associated shifts are the phenotypes that experimental osteoporosis models are designed to capture. These models remain the standard platform for linking altered bone remodeling to defined cellular and molecular perturbations (1). Why macrophages adopt one polarization state over another, and how that choice is imposed at the molecular level, cannot be answered by cell-surface phenotyping alone. Answering it means stepping inside the cell, to the metabolic programs that set and sustain each activation state.

## Metabolic reprogramming as an upstream regulator biasing macrophage polarization

Macrophage polarization has traditionally been framed as a response to extracellular cues. In this view, interferon-γ and lipopolysaccharide instruct a pro-inflammatory M1 state, while interleukin-4 and interleukin-13 drive a reparative M2 state. This cytokine-centric view, however, overlooks a more fundamental determinant. The intracellular metabolic program a macrophage adopts is not merely a consequence of activation. It is an active, upstream event that sets and sustains the functional phenotype (7). This immunometabolic principle is now codified as a foundational tenet of modern immunology (22). Distinct activation states are built on distinct metabolic architectures (23): inflammatory macrophages rewire toward aerobic glycolysis, while reparative macrophages depend on oxidative phosphorylation (OXPHOS) and fatty-acid oxidation (FAO). Manipulating these pathways is sufficient to redirect polarization independently of the initiating stimulus. Positioning metabolism upstream of phenotype recasts the central question of bone immunology: not “which macrophages are present” but “which metabolic state they occupy.” That distinction carries direct mechanistic and therapeutic consequences for osteoporosis.

The glycolytic axis is the best-defined route to the pro-inflammatory M1 phenotype. Upon inflammatory activation, macrophages shift from mitochondrial respiration to aerobic glycolysis, even under normoxia. This generates rapid ATP and biosynthetic intermediates that support cytokine production and effector function (7). The transition is driven largely by hypoxia-inducible factor 1-alpha (HIF-1α), which stabilizes under inflammatory, low-oxygen conditions to transactivate glycolytic enzymes and lock in the M1 program. Upstream receptors act on this switch. CXCR4 signaling reprograms macrophage metabolism toward glycolysis and thereby reinforces M1 polarization in a fibrotic context, linking a surface chemokine receptor to a defined metabolic-phenotypic outcome (24). At the enzymatic level, the glycolytic kinase PGK1 directly promotes M1 polarization and downstream inflammatory tissue damage. Specific glycolytic nodes, not just overall flux, are therefore causally coupled to phenotype (25). In bone the connection is demonstrable *in vivo*: pharmacological modulation of the HIF-1α axis in macrophages reshapes their polarization and alters alveolar bone resorption in a periodontal model (26). Across these studies, engagement of the glycolysis–HIF-1α module emerges as a proximate cause of the inflammatory, osteoclastogenic-permissive macrophage state, rather than a passive metabolic footprint of it.

The reparative M2 phenotype is sustained by a metabolically opposite program built on OXPHOS and FAO. Alternative activation depends on the capacity to oxidize fatty acids and fuel mitochondrial respiration. Lipid metabolism therefore positively regulates M2 commitment. Arachidonic-acid metabolism controls alternative activation specifically by regulating oxidative metabolism, a mechanistic bridge between a defined lipid substrate and the M2 program (27). The rate-limiting FAO enzyme carnitine palmitoyltransferase 1A (CPT1A) reinforces this axis: CPT1A-dependent metabolic rewiring drives an IL-10–associated phenotypic shift that dampens inflammation and favors tissue repair (28). Lipid-handling signals extend beyond intracellular oxidation. Apolipoprotein E expressed by macrophages transmits immunometabolic signaling that shapes activation state and the surrounding lipid environment (29). Conversely, mitochondrial dysfunction biases macrophages toward the pro-inflammatory M1 pole (30). The oxidative competence lost in that setting is the very capacity on which the reparative program depends (31). This reciprocity is the crux of the switch. A single metabolic decision favors OXPHOS/FAO and the M2 phenotype while it withdraws support from the glycolytic M1 program. Still, casting this axis as a bistable switch would inflate the underlying biology. Glycolysis and oxidative phosphorylation are not exclusive opposites: inflammatory macrophages preserve their mitochondrial content and keep oxidizing substrates, reparative macrophages maintain a detectable glycolytic flux, and transcriptome-wide profiling of human macrophages challenged with a broad stimulus panel resolves a continuous spectrum of programs rather than a pair of attractor states (90). The axis is thus more aptly characterized as a graded, reversible bias whose set-point is perpetually re-tuned by substrate availability, oxygen tension, mechanical loading and signaling history, and within which individual cells settle at intermediate or mixed positions. When this review speaks of a metabolic “switch,” it points to a displacement of that balance and a change in the relative dominance of one program over the other, not to a discrete two-state toggle (**Figure 2**).

**Figure 2:**
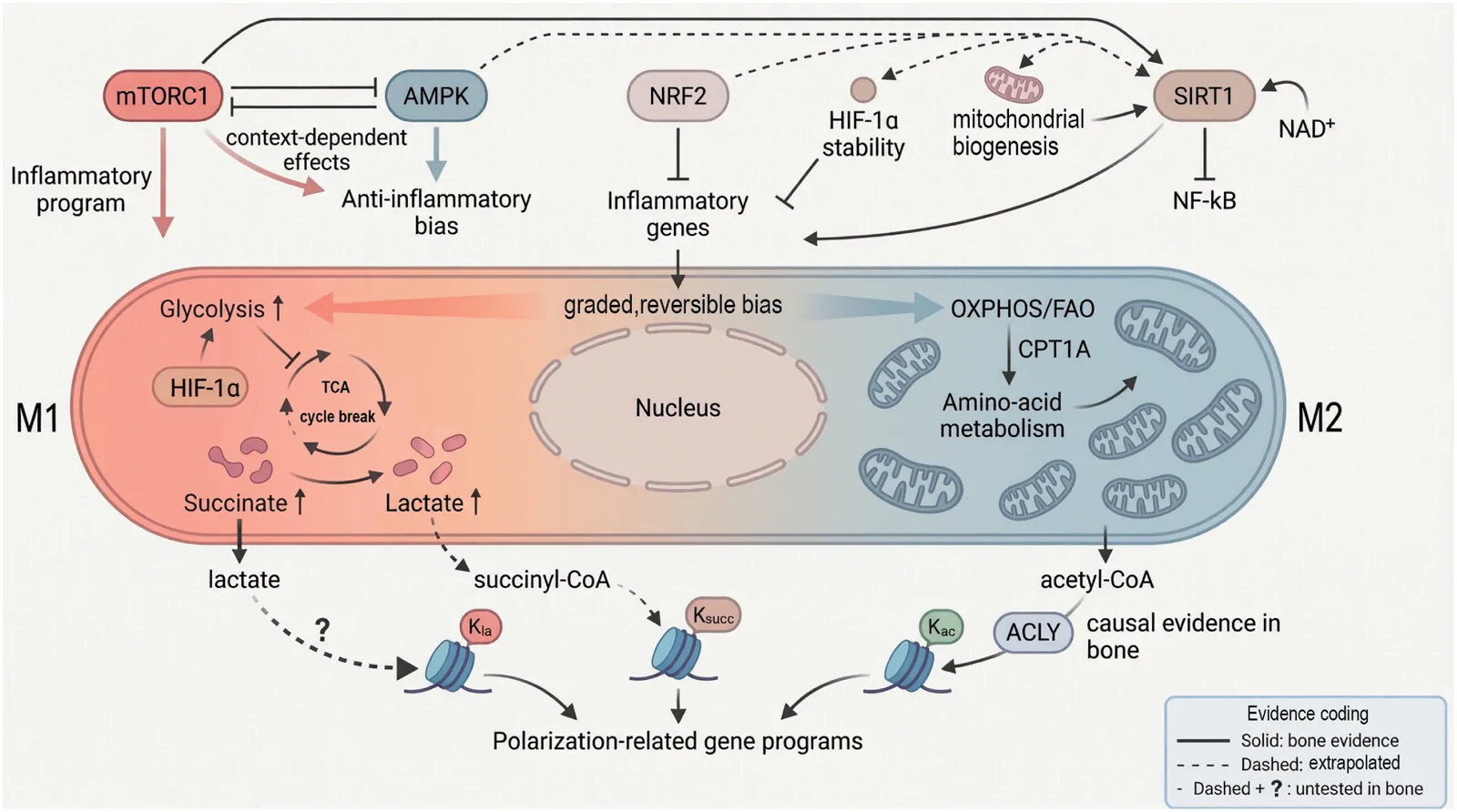
Metabolic switches driving macrophage polarization and their epigenetic output in bone. A macrophage cross-section is divided into pro-inflammatory (M1, left) and pro-resolving (M2, right) programs. The M1 side shows enhanced glycolysis, HIF-1α stabilization, a broken TCA cycle, and accumulation of succinate and lactate; the M2 side shows oxidative phosphorylation and fatty-acid oxidation (CPT1A) together with amino-acid metabolism. Centrally, lactate feeds histone lactylation, providing a metabolite → post-translational-modification → gene-program bridge that feeds back onto polarization genes to close the loop. An upper regulatory tier depicts the sensors that set where a macrophage sits along this gradient: mTOR complex 1 and AMPK as reciprocal nutrient- and energy-sensing inputs, NRF2 as the redox-responsive transcriptional brake on inflammatory gene expression, and SIRT1 as the NAD+-dependent deacetylase that restrains inflammatory signaling, each drawn converging on HIF-1α stability and mitochondrial biogenesis rather than acting in parallel to them. Additional metabolite-derived modifications are shown alongside lactylation, with succinyl-CoA-driven succinylation arising from the interrupted tricarboxylic-acid cycle and acetyl-CoA-driven acetylation arising from oxidative and lipogenic flux, so that the three marks are represented as divergent read-outs of the same metabolic shift. The figure highlights lactylation as the proposed metabolic–epigenetic link and represents the glycolysis/OXPHOS balance as a graded, reversible bias rather than a two-state switch; broken outlines and question marks denote steps for which no measurement in bone-resident macrophages is yet available, including macrophage lactylation within bone.

Framing the axis through HIF-1α and CPT1A alone also belittles the regulatory architecture that fixes a macrophage’s position on this gradient; four additional nodes merit explicit attention. mTOR complex 1 couples nutrient and growth-factor sensing to anabolic commitment and is an established regulator of skeletal development and disease, active in both the osteoblast and osteoclast lineages, which makes it a shared control point linking the immune and structural compartments of bone (97); its hyperactivation is likewise the route by which senescent stromal cells maintain an inflammatory secretory program during inflammatory bone loss (82). AMP-activated protein kinase serves as the counterposed energy sensor and steers macrophage polarization toward an anti-inflammatory functional state, so that the AMPK–mTOR balance constitutes a bidirectional rheostat on inflammatory bias (98). NRF2 couples redox state to transcription and restrains the macrophage inflammatory response by repressing proinflammatory cytokine transcription directly at the chromatin level, not merely by scavenging reactive oxygen species (99) — a mechanism with specific relevance to bone, since the resorbing environment is oxidatively stressed. NAD^+^-dependent deacetylation by SIRT1 curtails inflammatory signaling in macrophages and tunes systemic insulin sensitivity (100), and myeloid-restricted deletion of SIRT1 is alone sufficient to trigger inflammatory signaling under environmental stress (101), making SIRT1 the clearest instance of a metabolic sensor whose loss confined to the macrophage lineage yields an inflammatory phenotype. Two caveats apply. These four nodes are not parallel substitutes for the glycolysis–oxidation axis but its upstream controllers: each funnels into the same downstream decision by calibrating HIF-1α stability, mitochondrial biogenesis, or acetyl-CoA and NAD^+^ availability. And, with the partial exception of mTOR, their specific characterization within bone-resident macrophages is lacking; the evidence cited here is drawn from adipose tissue, liver, lung and peritoneal macrophages, and its skeletal relevance is, at present, an extrapolation of the sort this Review flags throughout.

These principles, largely established in non-skeletal tissues, are now being validated within the bone microenvironment, where they acquire direct relevance to osteoporosis. Immunometabolism of macrophages in the bone microenvironment has been articulated as a distinct organizing framework for bone healing and homeostasis (8). It explicitly transplants the metabolism-first logic into skeletal biology. Whereas that account is oriented to repair and regeneration, the present review extends the metabolism-first logic in a different direction. It traces that logic as a continuous causal cascade into chronic osteoporotic bone loss and its metabolic comorbidities. Functional consequences are beginning to be demonstrated directly in bone. In a recent model, macrophage metabolic reprogramming via the HIF-1α–glycolysis axis was shown to drive osteoblast ferroptosis and consequent bone loss. This single study delineates a causal chain from a macrophage metabolic state to a skeletal deficit (32). Parallel work in cartilage and joint tissue reinforces the generality of the HIF-1α–glycolysis module in musculoskeletal pathology, where hypoxic metabolic reprogramming reshapes the local inflammatory environment (33). Even mechanical inputs converge on this metabolic node. The mechanosensor PIEZO1 governs glycolysis in the joint microenvironment, indicating that biomechanical signals, a defining feature of bone, feed into the same metabolic circuitry that shapes the local inflammatory environment (34). Direct skeletal evidence is accumulating within the osteoclast lineage itself, where metabolic reprogramming is now recognized as an intrinsic feature of differentiation (35). Three findings illustrate this. The demethylase LSD1 metabolically integrates osteoclast differentiation and inflammatory bone resorption through HIF-1α (36). PRMT6-driven epigenetic control shifts osteoclast precursors from fatty-acid oxidation toward glycolysis to promote differentiation during osteoporosis (37). And glutaminolysis supplies the nucleotides and amino acids that osteoclast differentiation requires (38). These bone-intrinsic findings mirror, in the resorptive lineage, the glycolysis-versus-oxidation logic established in other tissues. Collectively, these studies move the metabolic-polarization coupling from a general immunological principle to a working hypothesis. They begin to show how bone-resident and bone-recruited monocytes tilt the remodeling balance toward loss (**Table 1**).

The therapeutic corollary is that metabolic nodes (HIF-1α, discrete glycolytic enzymes, and FAO regulators such as CPT1A) represent tractable, upstream intervention points. Rather than merely neutralize a single cytokine, they could reset polarization at its source. This logic is already being pursued in metabolic disease. Reprogramming of macrophage metabolism is emerging as a therapeutic axis in obesity, a condition mechanistically and epidemiologically intertwined with bone loss (39). Yet an honest assessment reveals a critical gap. Much of the metabolism-polarization evidence remains borrowed from lung, tumor, cardiovascular, and adipose contexts, and direct demonstrations within osteoporotic bone are still sparse. This caveat surfaces at every mechanistic tier of the Review and caps the strength of the claims that follow; the underlying arithmetic is worth laying out plainly. Among studies that tie a specified macrophage or osteoclast-lineage metabolic node to a skeletal readout, only a minority were carried out in a primary osteoporosis model: the PRMT6-driven skewing of osteoclast precursors from fatty-acid oxidation toward glycolysis during osteoporosis (37), the macrophage-senescence work in age-related and D-galactose-induced bone loss (80, 81), and the immunosenescence–grancalcin axis of skeletal ageing (78). A comparable evidence base comes from inflammatory or periodontal bone loss, where the HIF-1α, LSD1 and GLUT1 findings were obtained (26, 36, 83), while a third cluster is wholly non-skeletal (24, 25, 27–29, 33, 34, 39). These three strata are not interchangeable. Inflammatory bone loss is propelled by an exogenous microbial or immune trigger and carries a cytokine milieu far more intense than that of estrogen-deficient or age-related bone loss, so a metabolic dependency established there may mirror the scale of the inflammatory input rather than a mechanism at work in primary osteoporosis. To keep this contrast explicit rather than implied, every entry in **Table 1** is now tagged by model class, and in the text that follows the unqualified word “osteoporosis” is reserved for findings from ovariectomy, ageing or glucocorticoid models, with the model named outright in all other instances. Broad syntheses of polarization and its therapeutic modulation continue to treat metabolism as one input among many rather than the organizing axis (40). The central unanswered questions are therefore skeletal-specific. Which metabolic switch dominates in bone-resident macrophages under estrogen-deficient or age-associated conditions? Is the HIF-1α–glycolysis axis necessary, as well as sufficient, for osteoclastogenic bias *in vivo*? And how do the bone niche’s unique features (hypoxia gradients, mechanical loading, mineral turnover) constrain a macrophage’s metabolic options? Addressing these questions is the prerequisite for translating metabolic reprogramming from a compelling principle into a bone-directed therapeutic strategy. It also motivates the metabolic-modification mechanisms examined in the next section.

## Lactylation and emerging metabolite-derived modifications

A caveat shapes this section as a whole, and we foreground it at the outset rather than reserving it for a closing qualification. To our knowledge, no study has directly quantified histone lactylation — or any other lactate-derived modification — in bone-resident macrophages or in osteoclast precursors within bone. The intra-osseous lactylation evidence available at the time of writing is restricted to two contexts, neither macrophage-derived: endothelium-produced lactate driving histone lactylation in bone-marrow mesenchymal stromal cells (51), and p300-mediated histone lactylation fostering osteoblast differentiation (52), alongside a third account in a mineralizing periodontal lineage (53). Every macrophage-specific lactylation result discussed below was secured outside the skeleton, in tumor, pulmonary, myocardial, muscle-regeneration or peritoneal-macrophage settings. This section is therefore offered as a hypothesis built on cross-tissue evidence: the proposition that lactylation constitutes the epigenetic step by which a glycolytic macrophage locks in its inflammatory phenotype in bone is mechanistically well grounded and, in our view, the most testable prediction this Review advances — but it remains a prediction, not an established skeletal mechanism. The causal arrows traced in this section should accordingly be read as hypotheses awaiting the measurements that would confirm them, and the therapeutic inferences drawn at the section’s close as correspondingly provisional. If the choice of metabolic pathway sets macrophage phenotype from upstream, the metabolites generated along the way are what make that choice durable. Rather than inert end-products, they act as signaling molecules that feed back onto the epigenome and consolidate phenotype. Lactate is the paradigmatic example. Long dismissed as a glycolytic waste product cleared to sustain redox balance, lactate is now recognized as the substrate for a distinct covalent post-translational modification, lactylation, that couples the metabolic state of a cell to its transcriptional program (41). This reframing matters for bone immunology because it supplies a candidate molecular mechanism by which the glycolytic bias of an inflammatory macrophage, described in Section 3, could be written into durable gene-expression changes rather than remaining a transient metabolic footprint. Positioned this way, lactylation is the proposed second tier of the metabolism–polarization coupling: metabolic flux selects a phenotype, and the resulting metabolite pool locks it in (42) (**Figure 2**).

The mechanistic foundation of this concept was laid by the demonstration that BCAP-dependent glycolysis generates the lactate pool required for histone lactylation in macrophages, directly linking innate signaling to chromatin remodeling (9). In that study, BCAP-deficient macrophages were shown to lose histone lactylation at homeostatic gene loci (e.g., Arg1, Klf4, Mmp9), which was restored by exogenous lactate supplementation. This established a “lactate-clock” that shifts macrophage gene expression during inflammatory activation. Subsequent chromatin-level analyses reinforced the regulatory weight of this mark: H3K18la preferentially decorates tissue-specific active enhancers and promoters, indicating that lactylation partitions with regulatory chromatin as a targeted epigenetic signal rather than a diffuse chemical byproduct (43). The mark is also wired into downstream gene-regulatory circuits beyond transcriptional activation itself; histone lactylation has been shown to upregulate the RNA m6A reader YTHDF2, thereby coupling lactate-driven chromatin remodeling to post-transcriptional control of transcript stability (44). These studies establish lactylation as a bona fide epigenetic axis, though its enzymology and site specificity remain incompletely mapped (45).

Beyond chromatin, lactylation reshapes macrophage activation through both histone and non-histone routes, providing multiple levers on the M1/M2 balance. On the metabolic-enzyme side, lactylation of the glycolytic kinase PKM2 restrains the pro-inflammatory program and promotes the metabolic adaptation that accompanies the transition away from an inflammatory state, revealing a negative-feedback loop in which the product of glycolysis modifies a glycolytic enzyme to temper inflammation (46). Lactate-driven lactylation of the alarmin HMGB1 in macrophages promotes its exosomal release, extending the reach of the modification beyond the cell of origin and into intercellular signaling (47). These specific mechanisms sit within a broader body of work establishing lactate and lactylation as central to macrophage metabolic reprogramming and to the resolution of inflammation (48). The functional relevance of histone lactylation across macrophage biology and disease has been synthesized in detail, consolidating the view that the mark tunes activation state rather than merely accompanying it (49). Much of the direct macrophage evidence, however, derives from non-skeletal disease models (for instance, lactylation-dependent control of macrophage activation demonstrated in pulmonary pathology), and its skeletal generalization is an inference that still requires testing (50).

Critically for the present review, the bone microenvironment has begun to furnish direct evidence that lactylation operates within skeletal cells. Endothelial-derived lactate has been shown to trigger histone lactylation in bone marrow mesenchymal stromal cells, coupling the metabolic output of the local vasculature to the epigenetic state of a bone-forming progenitor population (51). The mark also operates in differentiated skeletal cells: lactate-induced, p300-mediated histone lactylation promotes osteoblast differentiation directly (52), and lactylation coupled to KDM6B-dependent demethylation regulates cementogenesis in a mineralizing periodontal lineage (53). These findings are pivotal because they transplant the lactate-to-chromatin mechanism from tumor and immune contexts into the bone niche itself, and they implicate the vascular compartment, a defining feature of the remodeling surface, as a source of the lactate signal. The emergence of lactylation as a theme in skeletal biology has prompted dedicated synthesis of its roles in bone health and disease (10). The present section deliberately narrows that broad survey to the macrophage-centered axis: rather than cataloguing lactylation across every bone cell type, it treats lactylation as the epigenetic consolidation step of the metabolism–polarization coupling, integrating it into the immunometabolic narrative that runs through this review. Worth restating is that the direct skeletal evidence is limited to stromal and osteoblast-lineage cells: in the endothelial study, lactylation was assayed in mesenchymal stromal cells (51), and in the differentiation study, in osteoblasts (52) — in neither instance was the modification probed in a macrophage. The macrophage arm of the case made here is thus, within bone, wholly unmeasured. This lacuna is not a trivial omission but the single most consequential gap in the framework, and it delineates a concrete, immediately tractable research frontier: mass-spectrometric or antibody-based quantification of pan-lysine and H3K18 lactylation in osteomacs and osteoclast precursors isolated from ovariectomized and aged bone, with and without pharmacological or genetic manipulation of glycolytic flux, would either validate this tier of the model or refute it.

The regulatory logic of lactylation also extends into systemic and tissue-repair processes, arguing for its breadth as a metabolic-signaling node rather than a niche phenomenon. In macrophage efferocytosis, glycolytic flux through PFKFB2 generates the lactate that drives the continued clearance of apoptotic cells, tying a specific glycolytic enzyme to a lactate-dependent reparative behavior with obvious relevance to the clearance demands of remodeling bone (54). In the infarcted myocardium, histone lactylation promotes the activation of reparative gene programs, offering a cross-tissue precedent for lactylation steering macrophages toward repair rather than inflammation (55). Endothelial lactate can instruct an M2-like, pro-regenerative macrophage phenotype in regenerating muscle, a mechanism structurally analogous to the endothelial-lactate signaling now documented in bone (56). At the organismal level, exercise-induced lactate and consequent lactylation have been implicated in the beneficial remodeling of metabolic disease, indicating that the lactate–lactylation axis is responsive to physiological inputs that also influence skeletal loading (57). These cross-tissue findings are mechanistic borrowings rather than direct skeletal proof, but their convergence on a repair-associated, M2-leaning output makes lactylation a plausible switch for the reparative arm of bone remodeling.

The broader lesson is that lactate is only the best-characterized member of a wider class of metabolite-derived protein modifications, in which a metabolic intermediate acts as a covalent substrate that writes cellular state onto histones and metabolic enzymes alike. Krebs-cycle-linked intermediates such as succinate and itaconate have themselves been reimagined as signal transducers that reprogram macrophage inflammation (58, 59). Itaconate, produced by the immunometabolic enzyme ACOD1 (60), can in turn target JAK1 to restrain alternative macrophage activation (61), and the shifting metabolic milieu generates further acylations, pointing toward a combinatorial code in which the metabolic environment is transcribed into a durable regulatory signature. It is illuminating to juxtapose the three best-characterized members of this class directly, for they diverge systematically in both the maturity of their evidence and their proximity to bone. Lysine acetylation is the oldest and best-validated of the three: its enzymology is well delineated, and it has a direct osteoclast-level demonstration in bone, given that inhibition of ATP-citrate lyase lowers nucleocytosolic acetyl-CoA and restrains osteoclast differentiation and function through diminished histone acetylation (103) — making acetylation the sole metabolite-derived modification with an established causal link to a skeletal cell-fate decision. Lysine succinylation was recognized more recently as a distinct modification governed by succinyl-CoA availability (102) and is appealing in principle because succinate accumulates precisely in the broken tricarboxylic-acid cycle of the inflammatory macrophage (58); we are not aware, however, of any study that has quantified succinylation in a macrophage or osteoclast within bone, so its role here is inferential in a still stronger sense than that of lactylation. Lactylation holds an intermediate position: mechanistically established in macrophages outside the skeleton (9, 46, 47) and established within bone in non-macrophage lineages (51, 52), but not yet in both at once. The three also differ in their driver — acetylation tracks acetyl-CoA and thereby oxidative and lipogenic flux, whereas lactylation and succinylation track glycolytic overflow and tricarboxylic-acid-cycle interruption, respectively — so an inflammatory metabolic shift should be anticipated to elevate the latter pair while depressing the former. To our knowledge, that prediction has never been tested in bone, and testing it would demand nothing more than parallel quantification of the three marks within the same sorted macrophage population. The therapeutic corollary is immediate: if metabolite-derived modifications consolidate macrophage phenotype, then the enzymes that write and read them become candidate targets for redirecting polarization. Whether that redirection ultimately protects the skeleton depends on what the fixed phenotype does to bone: the balance of resorption and formation that the next section takes up.

## The immunometabolic axis proposed to modulate bone resorption and formation

Metabolic pathway selection and metabolite-derived modifications fix macrophage phenotype, but the decisive question for osteoporosis is what that fixed phenotype does to bone. The terminal variable is not polarization per se but the net balance of remodeling: whether resorption by osteoclasts outpaces formation by osteoblasts. This section translates the metabolism–polarization coupling into that balance, arguing that a reprogrammed, inflammatory-leaning macrophage compartment simultaneously drives the resorptive arm and erodes the formative and vascular support of bone, tipping the equilibrium toward loss. Narrative reviews have repeatedly linked macrophage polarization to osteoporosis (11, 12), yet they tend to treat polarization as an endpoint; here it is treated as the input to a mechanistic ledger whose output is measured in bone mass. Positioning the section this way keeps the upstream metabolic detail of Sections 3 and 4 in the background and foregrounds the skeletal consequences that make immunometabolism clinically consequential.

The resorptive arm is engaged first through inflammatory signaling nodes that couple mitochondrial quality control to inflammasome activation. In inflammatory bone loss, a STAT3-dependent mitophagy axis coordinates NLRP3 inflammasome activity, so that failure to clear damaged mitochondria permits the inflammasome and amplifies the osteoclastogenic cytokine milieu (62). This links the mitochondrial state emphasized in Section 3 to a concrete downstream effector: defective mitophagy acts as a permissive signal for the IL-1β-driven program that favors osteoclast differentiation. The therapeutic mirror image reinforces the causal logic, as driving mitophagy through APJ activation attenuates systemic inflammatory bone loss, indicating that restoring mitochondrial turnover in the macrophage compartment is sufficient to blunt resorption *in vivo* (63). Read together, these studies frame mitochondrial quality control as a rheostat on the resorptive arm: when clearance fails, the inflammasome-osteoclast axis runs unchecked; when it is maintained, bone loss is contained. The broader role of autophagy and mitophagy in bone metabolic disorders situates this mitochondrial-clearance axis within an established skeletal regulatory theme (64).

A second set of controls operates at the canonical signaling nodes of osteoclast commitment, where the RANKL–TRAF6 relay is tuned by degradation and membrane-organization mechanisms. TRAF6 is the obligate adaptor transducing RANKL into the osteoclastogenic transcriptional program, and its abundance is rate-limiting: proteasomal control of TRAF6 by the REGγ system maintains bone homeostasis, and loss of that control predisposes to osteoporotic bone loss by allowing unchecked osteoclast differentiation (65). Membrane microdomains provide a parallel layer of regulation. Targeting the lipid-raft scaffold protein stomatin ameliorates osteoporosis, implicating raft-dependent organization of receptor signaling in setting the osteoclastogenic threshold (66). These findings matter for the immunometabolic thesis because both degradation flux and lipid-raft composition are themselves metabolically sensitive: proteostatic capacity and membrane lipid supply are downstream of the very metabolic reprogramming described earlier. The signaling node is thus not a fixed switch but a metabolically responsive valve on the resorptive arm.

Crucially, the same macrophage-lineage decisions that feed osteoclastogenesis also govern the vascular and formative side of the ledger, so that a single polarization bias can depress bone formation while it amplifies resorption. Bone-marrow macrophage differentiation has been shown to mediate both osteoclast generation and the formation of type H vessels through regulators such as RKIP, directly coupling the myeloid compartment to the angiogenic niche that supports osteogenesis (67) (**Figure 3**). Type H vasculature is the structural conduit linking angiogenesis to bone formation, and osteoblast-lineage-derived VEGF is a defining coupling signal that sustains this angiogenic–osteogenic axis (68). Bone formation is moreover energetically demanding in its own right (the osteoblast’s reliance on glucose and its wider energy metabolism carry direct implications for osteoporosis (69, 70)), so a macrophage-driven shift in the local metabolic environment can constrain the formative arm bioenergetically, not only through withdrawal of vascular support. The implication is that an inflammatory macrophage program does double damage: it enlarges the osteoclast pool and, by skewing marrow differentiation away from vessel-supportive phenotypes, degrades the type H vascular scaffold on which new bone depends. Resorption and impaired formation are thus two outputs of a single upstream immunometabolic decision rather than independent lesions, and this shared origin explains why polarization state maps so tightly onto net bone loss.

**Figure 3:**
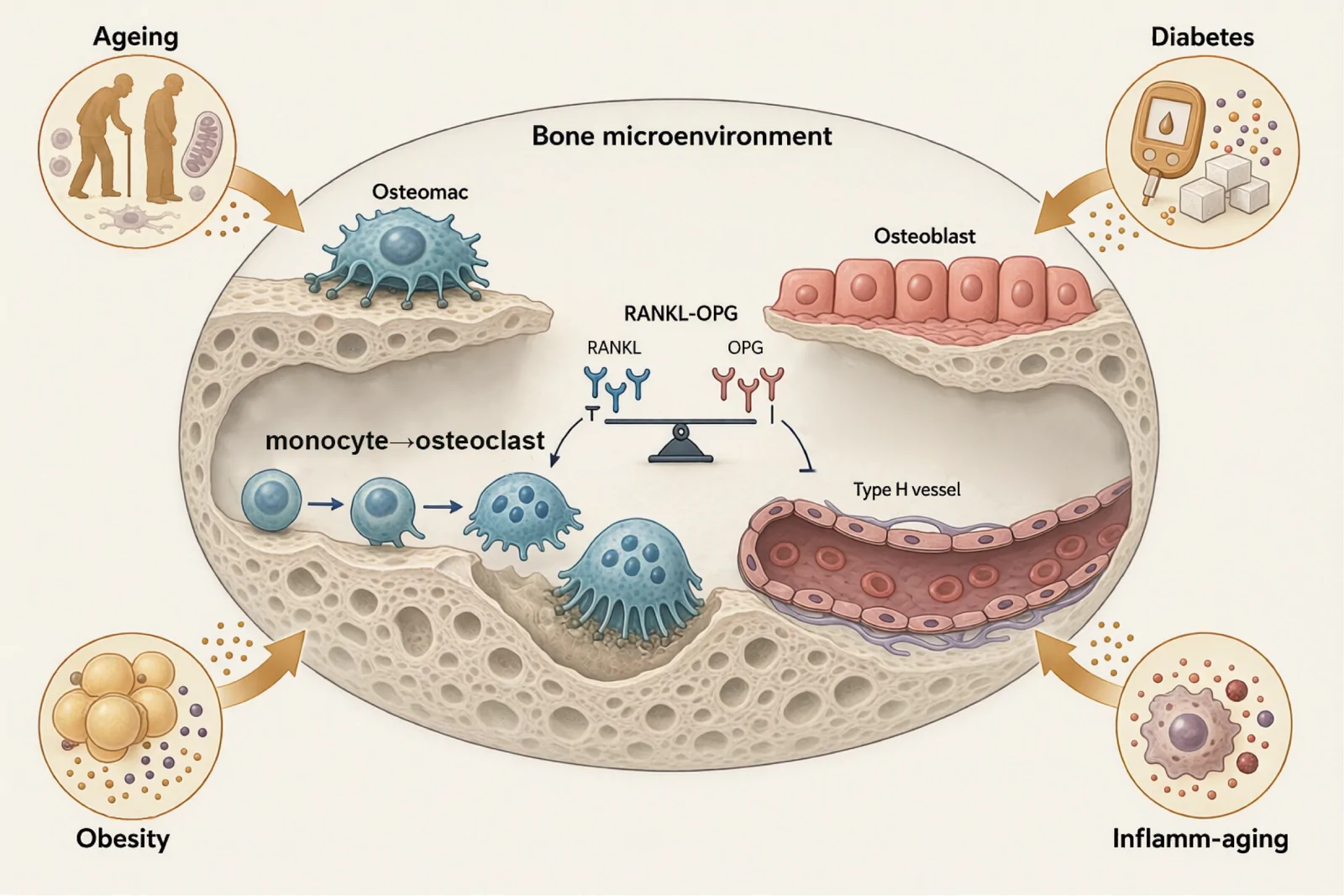
The immunometabolic axis in the bone microenvironment and its amplification by metabolic commodities. A trabecular bone-surface scene integrates cellular cross-talk and spatial organization: bone-resident osteomacs apposed to the bone surface, the monocyte-to-osteoclast lineage, osteoblasts, type H vessels, and the RANKL–OPG ligand–decoy axis. Peripheral inward arrows depict metabolic comorbidities (ageing, diabetes, obesity, and inflammaging) converging on the central microenvironment as amplifiers, with the microenvironment at the center and comorbidity amplifiers at the corners. The figure emphasizes that spatial proximity and comorbidity-associated metabolic load jointly tune the RANKL–OPG balance and the prevailing polarization tone.

If the inflammatory arm tips the balance toward loss, the reparative M2 arm can pull it back, and this reversibility is what renders the axis therapeutically addressable. M2 macrophages restrain the pathological program through both cellular and vesicular routes: M2-derived extracellular vesicles carrying glutamate alleviate osteoporosis, demonstrating that a specific M2 metabolite cargo can be transferred to skeletal targets to protect bone (71). The principle of inter-tissue metabolic transfer into bone extends beyond immune cells, as skeletal muscle-derived vesicles can carry glycolytic enzymes to the skeletal compartment, illustrating that metabolic machinery itself is trafficked to bone (72). The reparative reach extends to other immune effectors, as M2-derived vesicles reprogram neutrophils, indicating that the M2 phenotype drives a broader anti-resorptive, pro-repair tissue response rather than acting cell-autonomously (73). Conversely, suppressing the inflammatory pole is protective, as induced mesenchymal stromal cells inhibit M1 macrophage polarization, shifting the balance away from the osteoclastogenic environment (74). These interventions share a logic: they either supply M2-associated signals or subtract M1-associated ones, and in each case the skeletal readout improves. The reparative arm thus operationalizes the metabolism–polarization coupling as a bidirectional dial, establishing that the same axis driving loss can, when redirected, drive gain.

Synthesizing across these strands closes the mechanistic loop that this review has built from metabolism to bone fate. Metabolic pathway selection (Section 3) and metabolite-derived modification (Section 4) set macrophage phenotype, and that phenotype is now shown to determine the resorption–formation balance through four routes: inflammasome-coupled osteoclastogenesis, metabolically tuned RANKL–TRAF6 signaling, myeloid control of the type H vascular niche, and a redirectable M2 reparative arm. One reservation is specific to this ledger: the coupling between a given metabolic perturbation and a defined osteoclast- or osteoblast-level outcome is often established through correlation or single-pathway manipulation, so the integrated, quantitative contribution of the macrophage compartment to net bone balance *in vivo* remains to be resolved. That open question does not weaken the architecture of the axis so much as define the measurements it now demands. With the cell-intrinsic circuit in place, the question becomes how systemic metabolic comorbidities such as ageing, diabetes, and obesity feed into this same axis to produce secondary bone loss.

## Metabolic comorbidities and secondary bone loss

The cell-intrinsic circuit built so far runs from metabolic pathway selection through polarization and its epigenetic consolidation to an imbalance in resorption and formation. That circuit does not operate in isolation. It is embedded in a systemic metabolic context. The most common conditions that accelerate bone loss in clinical practice, namely ageing, diabetes, and obesity, share a single upstream feature: a chronic, low-grade inflammatory state that reprograms macrophages toward the phenotype the earlier sections identified as osteo-catabolic. The concept of “inflamm-aging,” the age-associated rise in systemic pro-inflammatory tone, provides the organizing frame for this section, having been proposed as a general driver of degenerative disease including skeletal decline (75). Osteoporosis has correspondingly been reframed, at least in part, as an inflammatory disorder in which immune activation contributes to net bone loss rather than acting as a bystander (76). Viewed through the immunometabolic lens developed here, these comorbidities are not separate diseases that happen to co-occur with osteoporosis; they are systemic amplifiers that feed the same macrophage-centered mechanism (**Figure 3**).

Ageing is the paradigmatic amplifier, and it acts in large part through the macrophage compartment itself. The senescence of immune and stromal cells within the ageing musculoskeletal system establishes a self-sustaining inflammatory environment that biases macrophage polarization and undermines the coupling between bone formation and resorption (77). A concrete effector mechanism has been defined in the immune compartment: senescent immune cells release grancalcin, which acts on the skeletal niche to promote bone ageing, linking immune-cell senescence directly to a diffusible signal that drives skeletal deterioration (78). The stromal side of this ageing program is equally implicated, with osteocytes recognized as central sensors and effectors of bone ageing whose dysfunction reshapes the remodeling environment (79). At the cell-intrinsic level, restraint of macrophage senescence is protective: signaling through the β-catenin/FOXO1 axis limits the acquisition of a senescent, pro-inflammatory macrophage phenotype, and its failure permits the accumulation of senescent macrophages that accompany bone loss (80). The reverse manipulation is instructive. Pharmacological attenuation of macrophage senescence with astragaloside mitigates both the inflammatory phenotype and the associated bone loss, providing a causal link between macrophage ageing and skeletal deterioration (81). The mTOR pathway sits close to the center of this process. Hyperactivated mTOR signaling drives a senescence-associated secretory program that intensifies tissue inflammation. This mechanism, demonstrated in stromal fibroblasts, is mechanistically relevant to the metabolic control of the senescent macrophage phenotype, though its direct skeletal role requires confirmation (82). Ageing thus operates as a chronic, cell-autonomous nudge toward the inflammatory, glycolytically biased macrophage that the mechanistic sections linked to enhanced osteoclastogenesis.

Diabetes superimposes a distinct but convergent metabolic insult through chronic hyperglycemia. Elevated glucose is not merely a fuel excess; it reprograms macrophage metabolism toward an inflammatory, prematurely aged state. High glucose acting through ROS overproduction drives macrophage inflammatory senescence, coupling the hyperglycemic environment directly to the pro-inflammatory, M1-skewed phenotype that favors resorption over formation (83). This provides a concrete molecular route by which diabetic bone disease (characterized clinically by increased fracture risk despite variable bone density) can be understood as an immunometabolic complication rather than a purely endocrine one. Two points must be made if this is to amount to more than an assertion of convergence. The first is that a specific metabolic node is pinpointed, and not merely a shared inflammatory tone: the hyperglycemia/ROS study assigns the lesion to high-glucose-driven reactive oxygen species overproduction in macrophages, demonstrates that ROS scavenging reverses the M1 polarization and ameliorates alveolar bone loss *in vivo*, and documents a bone readout within the same system (83) — the minimum evidence required to situate a comorbidity on, rather than alongside, the macrophage axis. Beyond this hyperglycemia/ROS axis, however, specificity attenuates rapidly; the broader literature linking diabetes to osteoporosis casts the relationship as an imbalance between inflammation and bone remodeling without resolving which cell type harbors the metabolic lesion (107). The second point is that competing, macrophage-independent mechanisms are well established and are not excluded by any of this evidence. Hyperglycemia and advanced glycation end-products injure the osteoblast and the collagen matrix directly, insulin and IGF-1 signaling deficits undermine bone formation independently of immune cells, and diabetic microangiopathy compromises the vascular supply to the remodeling surface. None of these calls for a reprogrammed macrophage. The honest reading of the evidence is therefore that macrophage glycolytic reprogramming is a demonstrated contributor in inflammatory and periodontal models of diabetic bone loss and a plausible contributor to diabetic skeletal fragility more broadly, but that its quantitative share of the phenotype has not been partitioned against these parallel routes, and that doing so requires the myeloid-restricted loss-of-function experiments this Review repeatedly identifies as missing. The mechanism aligns with the glycolytic bias described in Section 3: where that section showed glycolytic flux selecting an inflammatory phenotype in principle, the diabetic setting supplies the systemic substrate excess that pushes the switch chronically in the same direction, sustaining the osteo-catabolic macrophage state over the long term.

Obesity and disordered lipid metabolism complete the triad, acting through both adipose-tissue inflammation and cell-intrinsic lipid handling within macrophages. Obesity is accompanied by extensive macrophage metabolic reprogramming, a process characterized in the cardiovascular context. That work establishes lipid overload as a driver of the inflammatory macrophage state, with clear relevance to the skeletal consequences of obesity (39). Central to this is the handling of cholesterol: intracellular cholesterol homeostasis is a decisive regulator of macrophage inflammatory output, such that its dysregulation under lipid excess biases activation toward the pro-inflammatory pole (84). The skeletal consequences of obesity, however, are considerably more heterogeneous than this mechanism alone would predict, and it would be misleading to cast obesity as uniformly osteoporotic. Higher body mass augments mechanical loading and the peripheral aromatization of androgens to estrogens, both of which favor bone accrual, and areal bone mineral density measured by dual-energy X-ray absorptiometry is frequently normal or elevated in obese individuals even as fracture risk at certain sites rises. Recent real-world densitometric data illustrate this dissociation directly: in postmenopausal women, body mass index correlates positively with bone mineral density yet negatively with trabecular bone score, so that mass is preserved while microarchitecture deteriorates (105). Reviews of obesity and bone metabolism accordingly portray a relationship that varies with fat distribution, marrow adiposity, skeletal site, sex and the metabolic health of the adipose tissue, rather than one with a single direction of effect (106). What the immunometabolic account illuminates, then, is not obesity-associated bone loss as such but a specific component of it — namely the inflammatory, lipid-laden macrophage state that accompanies metabolically unhealthy adiposity and that would be anticipated to degrade bone quality while leaving bone mass comparatively intact. Framed this way, obesity converges on the same macrophage-mediated mechanism as ageing and diabetes with respect to inflammatory tone and bone quality, whereas its net effect on bone mass reflects the sum of that mechanism and the mechanical and endocrine effects that oppose it. That three of the most prevalent chronic conditions in ageing populations feed a shared immunometabolic circuit helps explain why they cluster with accelerated bone loss, and why they may prove tractable to interventions aimed at that circuit.

One qualification is specific to this triad: these systemic states rarely occur in isolation in the ageing patient, and their interactions (hyperglycemia superimposed on inflamm-aging, obesity accelerating both) are likely to be non-additive in ways that current single-comorbidity models do not capture. What the immunometabolic framework contributes is a unifying hypothesis: that ageing, diabetes, and obesity share a final common pathway in the reprogrammed, inflammation-biased macrophage. That hypothesis reframes the comorbidities as amplifiers of one mechanism, and it raises a direct therapeutic question: whether the macrophage immunometabolic axis can be targeted to interrupt bone loss across these overlapping conditions.

A more fundamental caveat concerns causal attribution. The preceding sections might be read as implying more than the evidence allows: observing that a comorbidity coincides with macrophage metabolic reprogramming, and that both coincide with bone loss, does not prove that the skeleton loses bone through the macrophage. Each condition possesses a well-defined, macrophage-independent path to the skeleton. In ageing, menopausal estrogen withdrawal acts directly on osteoblasts, osteoclasts and osteocytes and remains the chief single driver of postmenopausal bone loss, while osteocyte dysfunction autonomously reshapes the remodeling milieu (79). In diabetes, glycation and impaired insulin signaling degrade matrix quality and formation, as detailed above. In obesity, marrow adipocyte expansion diverts a shared progenitor pool away from osteoblastogenesis (13). For none of the three has the macrophage-metabolic contribution been disentangled from these parallel pathways. The experiments needed to do so are well defined and, in our view, represent the highest-priority tests of this framework: bone-loss phenotypes after ovariectomy, ageing, diabetes or diet-induced obesity, assessed in mice carrying myeloid-restricted deletion of a core metabolic node — Hif1a, Cpt1a, Slc2a1 or Sirt1 — with rescue through a node-selective intervention. Two of the studies cited above begin to approach this design by manipulating a macrophage-level variable and reading out bone (81, 83), yet both rely on pharmacology or inflammatory models rather than lineage-restricted genetics in primary osteoporosis. Until such evidence is available, the defensible claim remains the narrower one advanced here: ageing, diabetes and obesity converge on a shared macrophage immunometabolic state; that state is sufficient to shift remodeling towards bone loss in the models tested; and whether it is necessary for the bone loss seen in each comorbidity is an open question.

## Therapeutic prospects of targeting macrophage immunometabolism

If metabolic pathway choice sets polarization, polarization is consolidated epigenetically, and the resulting phenotype tilts the resorption–formation balance, then each node in that sequence is a potential point of intervention. The established anti-resorptive pharmacology of osteoporosis does not exploit this. Agents such as the bisphosphonate clodronate act on the terminal osteoclast, suppressing bone resorption by impairing mature resorbing cells rather than by addressing the upstream immunometabolic program that generates and enables them (85). This distinction defines both the limitation of current therapy and the opening for an immunometabolic strategy: rather than silencing the effector cell after it has committed, intervening at the level of macrophage metabolism and polarization could recalibrate the balance before osteoclastogenesis is amplified, while preserving the reparative, osteogenesis-supporting arm of the macrophage response. The sections above frame the M2/reparative program as an endogenous physiological brake; the question this section addresses is how that brake might be engaged exogenously. Unlike syntheses that survey macrophage-targeted intervention for bone healing and regeneration, the strategies assessed here are evaluated specifically for their capacity to interrupt chronic osteoporotic loss and the secondary bone loss of its metabolic comorbidities (**Figure 4**).

**Figure 4:**
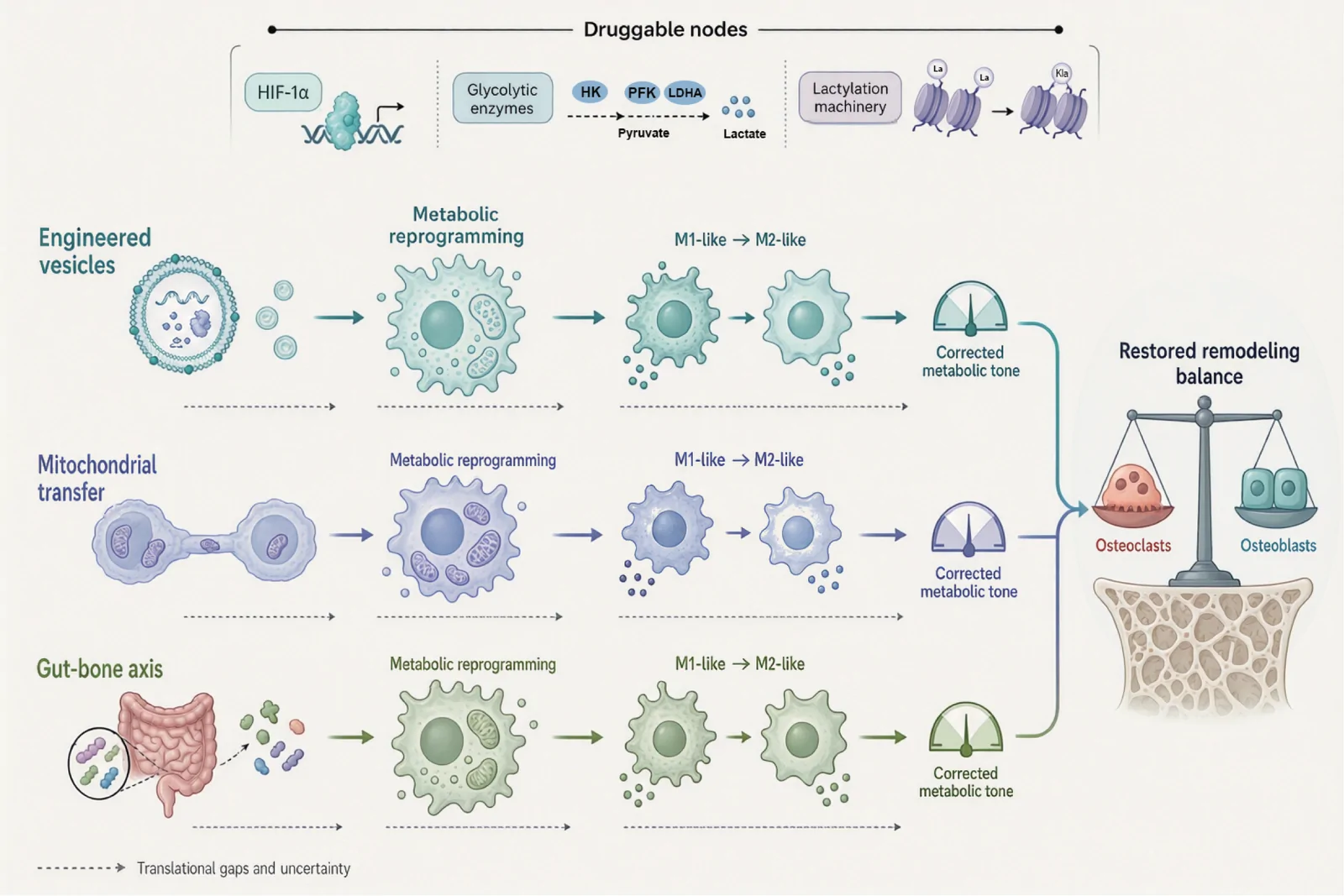
Targeting macrophage immunometabolism: a translational roadmap of therapeutic strategies. Three parallel lanes present therapeutic strategies: engineered vesicles, mitochondrial transfer, and the gut–bone axis. Each lane proceeds from target to reprogramming effect (M1→M2 repolarization or metabolic correction) to improved skeletal phenotype, with druggable nodes (HIF-1α, glycolytic enzymes, and the lactylation machinery) labeled along the way. Mechanistic anchors at the top refer back to **Figures 2**, **3**, and all lanes converge on the right onto restored remodeling balance, echoing the balance motif of **Figure 1**.

The most developed line of preclinical evidence targets polarization directly through engineered extracellular vesicles. Because macrophage-derived vesicles carry the functional cargo of their parent phenotype, reprogramming the vesicle, or the cell that produces it, offers a route to impose a pro-osteogenic, anti-osteoclastic environment on bone. Melatonin-engineered M2-like exosomes have been used, in a periodontitis model, as a mediator of therapy, exploiting the anti-inflammatory and phenotype-stabilizing properties of the cargo to steer the local macrophage response toward repair (86). In a mechanistically convergent approach, M2 macrophage-derived vesicles delivering glutamate have been shown to alleviate osteoporosis, illustrating that the therapeutic payload can itself be a metabolic intermediate rather than a conventional drug (71). These strategies are attractive because they co-opt an endogenous signaling modality and because vesicle engineering allows both the phenotype-instructing signal and a targeting capacity to be built into a single agent. They remain, however, proof-of-concept demonstrations in animal and cellular models, with the standard translational hurdles of vesicle manufacturing, dosing, biodistribution, and long-term safety unresolved.

A second and more recent frontier intervenes at the organelle and metabolic level rather than at the level of secreted signals. Since mitochondrial function is the pivot between the glycolytic, pro-inflammatory state and the oxidative, reparative state described in Section 3, directly repairing or replacing the metabolic machinery of a macrophage is a logical, if technically demanding, therapeutic concept. CXCR4-engineered macrophages have been used to achieve targeted mitochondrial transfer that reprograms recipient cells, coupling a homing modification that directs the carrier to the relevant niche with an organelle-level payload that rewrites metabolic state (87). This approach is conceptually distinct from the vesicle strategies above: instead of instructing polarization through a signaling cargo, it supplies the oxidative capacity that underlies the reparative phenotype, addressing the metabolic deficit at its source. As an emerging platform it sits at the earliest stage of the translational pipeline, and its skeletal application is an extension of a broader reprogramming principle rather than an osteoporosis-validated therapy, but it exemplifies the direction in which immunometabolic intervention is moving.

The final tier of intervention operates systemically, acting on the metabolic inputs that shape macrophage phenotype rather than on bone-resident macrophages directly. Colon-targeted postbiotics acting through the gut–bone axis have been shown to alleviate osteoporosis, providing evidence that a systemic, microbiome-linked input can be harnessed to shift the immunometabolic balance in favor of bone preservation (88). This axis-level strategy is appealing for its non-invasiveness and its alignment with the comorbidity biology of the preceding section, but it also carries the greatest mechanistic distance between intervention and skeletal endpoint, and the causal steps linking a gut-directed agent to macrophage reprogramming in bone remain to be resolved.

## Translational hurdles: delivery, targeting specificity and long-term safety

Three translational barriers span all three tiers; because they are often noted only in passing, we lay them out explicitly here.

The first is delivery. Systemically administered engineered vesicles are cleared rapidly by the liver and spleen, and only a small fraction reaches trabecular bone. The periodontal and local-defect contexts in which vesicle therapy has proved most convincing (86) allow local application — an option that chronic systemic osteoporosis does not afford. Manufacturing adds further difficulty: the isolation method dictates cargo composition, potency assays for a mixed-cargo biologic remain unsettled, and batch-to-batch consistency at clinical scale has not been demonstrated for any macrophage-derived vesicle product. Mitochondrial transfer is still more demanding: a mitochondrion is neither stable nor storable as a payload, must arrive in the recipient-cell cytoplasm intact and respiration-competent, and in the sole skeletal demonstration to date was delivered by an engineered carrier cell whose biodistribution, persistence and fate become intrinsic to the therapeutic product (87). Gut-directed postbiotics present the inverse problem — oral delivery is straightforward, but the agent acts several steps upstream of bone, so the administered dose bears no fixed relationship to the effect at the remodeling surface (88).

The second barrier, targeting specificity, has two readily conflated components. Tissue specificity means reaching bone rather than other organs; cell specificity means acting on bone-resident macrophages rather than the many other phagocytes, osteoblasts, stromal cells and endothelial cells that share the niche. Vesicle and nanoparticle platforms are avidly taken up by mononuclear phagocytes throughout the body, so a macrophage-reprogramming agent is, by default, a systemic macrophage-reprogramming agent. This matters for two reasons. First, the same polarization shift that protects bone would be expected to blunt antimicrobial and antitumor macrophage function elsewhere. Second, the metabolic nodes in question are not macrophage-restricted: HIF-1α, mTOR, SIRT1 and CPT1A are essential in hepatocytes, cardiomyocytes and hematopoietic progenitors. Receptor-directed homing offers a partial remedy. The CXCR4-engineered carrier used for mitochondrial transfer is an instructive precedent, because the homing modification and the payload were engineered together (87) — yet CXCR4 ligation itself reprograms macrophage metabolism toward glycolysis (24), so the targeting moiety is not pharmacologically inert. Any credible development path must therefore document both where the agent goes and which cells it acts on; the studies available at present report neither in bone.

The third barrier, long-term safety, carries greater weight in osteoporosis than in the acute indications for which these platforms were originally developed. Osteoporosis demands years of therapy, yet every study cited here administered the intervention for days to weeks. Chronic suppression of the inflammatory macrophage program raises the prospect of impaired infection control and diminished tumor surveillance; chronic enforcement of a reparative program carries the converse risk of fibrosis and impaired clearance of apoptotic cells — a function that itself depends on glycolytic flux (54). Sustained inhibition of glycolysis or HIF-1α would be expected to disrupt hematopoiesis, because the marrow is hypoxic and its progenitors are glycolysis-dependent — and the marrow is precisely the compartment the therapy must enter. Repeated administration of engineered vesicles or allogeneic carrier cells raises immunogenicity questions that no bone study has addressed, and microbiome-directed interventions risk durable, unintended shifts in a community whose composition shapes immunity far beyond bone. None of these risks is prohibitive, but none has been evaluated, and their absence from the current literature measures how early this field is rather than evidencing its safety.

## Bone-targeted delivery strategies

These obstacles converge on a single imperative: the metabolic targets identified in this review cannot be pursued systemically. HIF-1α illustrates why with unusual clarity. In macrophages, HIF-1α stabilization drives the glycolytic, inflammatory program that favors osteoclastogenesis (28, 32), and pharmacological modulation of this axis alters alveolar bone resorption *in vivo* (26). Yet in the osteoblast lineage and the skeletal vasculature, the same pathway is osteoanabolic: HIF-α signaling couples angiogenesis to osteogenesis during skeletal development, and its activation raises bone mass by expanding the vascular network that supports bone formation (104). The type H vessels on which formation depends are themselves HIF-responsive structures (67, 68). A systemically delivered HIF-1α inhibitor would therefore antagonize the macrophage-driven resorptive arm and the vascular–osteogenic formative arm at once, and the net skeletal outcome could plausibly be neutral or even deleterious. The same reasoning extends to the remaining nodes: CPT1A inhibition would suppress the reparative macrophage program that this review argues should be preserved; mTOR inhibition pushes osteoblasts and osteoclasts in opposite directions (97); and SIRT1 activation carries systemic metabolic consequences that reach far beyond bone (100). Cell-type-selective — and ideally bone-restricted — intervention is thus not a refinement of this therapeutic strategy but a precondition for it.

Three approaches to this problem exist, at sharply different stages of maturity. The most mature is chemical bone targeting, which leverages the affinity of certain moieties for hydroxyapatite. Bisphosphonate and tetracycline conjugation directs nanoparticles to mineralized surfaces; tetracycline-grafted poly(lactic-co-glycolic acid) nanoparticles have been characterized as a bone-targeting delivery platform (109), and a bone-targeting gene-delivery system carrying Semaphorin 3A ameliorated bone loss in ovariectomized mice, showing that the approach can deliver a biologically active payload to osteoporotic bone and alter the skeletal phenotype (108). Aspartate-rich oligopeptides and other acidic-motif ligands offer a comparable strategy, with preferential binding to resorptive rather than formative surfaces. The limitation shared by all mineral-affinity approaches is that they target matrix, not cells, so the payload must still be released and taken up by the intended macrophage; combining a mineral-binding ligand with a myeloid-directed carrier is the obvious next step and, to our knowledge, has not been reported in the skeletal immunometabolism literature. The second approach is receptor-directed cellular homing, exemplified by the CXCR4-engineered macrophage carrier used for mitochondrial transfer to the marrow (87); mannose- and folate-receptor-directed carriers achieve macrophage selectivity in other tissues but confer no bone specificity, and, as noted, the homing receptor may itself be metabolically active. The third is not a delivery strategy at all but its genetic equivalent, and it is the most urgent: myeloid- and osteoclast-lineage-restricted conditional deletion — using LysM-Cre, Cx3cr1-Cre or Ctsk-Cre drivers — of Hif1a, Cpt1a, Slc2a1 or the lactylation machinery, with bone mass and microarchitecture as primary endpoints. Such experiments would establish whether the macrophage node is necessary for bone loss and would simultaneously define the therapeutic window that a targeted agent must achieve. Their absence is, in our assessment, the principal reason this framework remains an emerging regulatory axis rather than a validated therapeutic strategy.

Rather than competing options, the three tiers are complementary entry points onto the same causal cascade: vesicle engineering acts on the polarization signal, mitochondrial transfer restores the metabolic capacity that underlies it, and gut–bone axis modulation adjusts the systemic inputs upstream of both. In principle they could be combined (a systemic input to lower the inflammatory set-point, a local signal to instruct phenotype, and an organelle-level payload to secure the reparative metabolic state), so that intervention is staged along the cascade rather than aimed at a single node. Against that promise a common translational reality holds: every strategy surveyed here remains preclinical, and the same *in vivo* bone-macrophage validation that this review has flagged throughout is the precondition for advancing any of them. What these approaches collectively establish is therefore not a ready therapy but a proof of principle: the metabolism–polarization coupling is druggable in multiple, mechanistically distinct ways (**Table 2**), and the task ahead is to convert that principle into interventions tested against the disease itself.

**Table 2:** Immunometabolic therapeutic targets and strategies in osteoporosis: mechanisms, evidence, and translational stage.

Strategy / target	Immunometabolic mechanism	Intervention modality	Evidence to date	Current development status	Major translational barriers	Supporting refs [ref no.]
Effector-level anti-resorptive — clodronate (incumbent comparator)	Acts on the terminal osteoclast; suppresses resorption without addressing the upstream immunometabolic program	Bisphosphonate (small molecule)	Established clinical anti-resorptive	Approved (comparator).	Effector-only mechanism: does not engage the upstream metabolic programme and does not preserve the reparative, osteogenesis-supporting macrophage arm; long-term adverse effects (atypical fracture, osteonecrosis of the jaw) with prolonged use	(85)
Melatonin-engineered M2-like exosomes	Anti-inflammatory, phenotype-stabilizing cargo steers local macrophages toward repair	Engineered extracellular vesicle	Animal / cellular (periodontitis therapy)	Preclinical proof-of-concept.	Rapid hepatic and splenic clearance after systemic dosing with minimal trabecular delivery; efficacy shown only with local application in a periodontal model; isolation-method-dependent cargo, absent potency assay and unproven batch consistency at scale; immunogenicity of repeated dosing unassessed	(86)
M2 macrophage-derived glutamate-loaded EVs	Metabolite (glutamate) cargo reshapes osteoclast-precursor fate	Metabolite-loaded extracellular vesicle	Animal (osteoporosis alleviated)	Preclinical.	Same vesicle manufacturing, dosing and biodistribution hurdles; no tissue or cell selectivity, so systemic phagocytes are reprogrammed alongside bone macrophages; duration of the metabolite-driven effect and dosing interval undefined	(71)
CXCR4-engineered macrophage mitochondrial transfer	Supplies oxidative capacity; reprograms recipient bone-marrow metabolism at the organelle level	Engineered cell / mitochondrial transfer	Animal (osteoporosis therapy)	Earliest translational stage.	Payload is neither stable nor storable and must arrive respiration-competent; the engineered carrier cell becomes part of the product, so its biodistribution, persistence and fate require separate characterization; the CXCR4 homing moiety itself reprograms macrophage metabolism and is not pharmacologically inert; allogeneic use raises immunogenicity questions	(87)
Gut–bone axis colon-targeted postbiotics	Systemic, microbiome-linked input shifts skeletal immunometabolic tone toward bone preservation	Colon-targeted postbiotic nanoparticle (systemic / oral)	Animal (osteoporosis alleviated)	Preclinical.	Greatest mechanistic distance to the skeletal endpoint, so delivered dose bears no fixed relationship to effect at the remodeling surface; the gut-agent to bone-macrophage steps are unresolved; durable and unintended microbiome shifts with systemic immune consequences	(88)
Upstream druggable metabolic nodes (HIF-1α, glycolytic enzymes, lactylation writers/readers)	Metabolic switches upstream of polarization; candidate points to recalibrate balance before osteoclastogenesis is amplified	Target / concept level (no dedicated bone-restricted agent yet)	Mechanistic validation in bone models (HIF-1α/DMOG; PRMT6; lactylation)	Conceptual / target-validation.	None of the nodes is macrophage-restricted, so systemic modulation affects hepatocytes, cardiomyocytes and haematopoietic progenitors; HIF-1α and mTOR act in opposite directions in macrophages versus the osteoblast–vascular compartment, so systemic inhibition may be self-defeating; no bone-restricted, macrophage-selective agent exists, and the required lineage-restricted knockout validation is absent	(26, 37, 51)

(Central challenge): all listed strategies are uniformly preclinical — none validated in a clinical osteoporosis population. The decisive hurdle is the spatiotemporal heterogeneity of the macrophage compartment; targeting specificity — not merely target validity — will determine success. Reference numbers in [] correspond to the numbered reference list.

## Conclusion and outlook

This review has framed macrophage immunometabolism as a metabolism–polarization coupling: the metabolic pathway a macrophage selects sets its polarization state. That state is then consolidated epigenetically through lactate-derived modifications and is ultimately translated into skeletal fate through the balance between osteoclastic resorption and osteogenic–vascular support. Cast this way, immunometabolic reprogramming emerges as a candidate upstream regulatory axis rather than a peripheral correlate of bone loss: a potentially actionable dimension of osteoporosis that lies beyond the terminal osteoclast at which current antiresorptive therapy is aimed. We restate, as a matter of scientific accounting rather than modesty, that this remains a framework to be tested. Its central mechanistic steps have been demonstrated in non-skeletal tissue and in inflammatory or periodontal bone-loss models; within primary postmenopausal or age-related osteoporosis they are, for the most part, inferred.

The value of this framework is that it converts a scattered set of observations into a single testable axis. It offers a common explanation for the secondary bone loss shared by ageing, diabetes and obesity; it recasts the sequence from metabolic node through polarization to bone fate as a set of experimental variables; and it identifies polarization and lactylation signatures as candidate readouts for patient stratification and for shifting therapy from suppressing mature osteoclasts toward reprogramming macrophage metabolism itself.

Realizing this potential requires confronting layered obstacles that are logically linked rather than independent. *In vivo* causal evidence remains thin: bone-macrophage-specific perturbation of the writing and reading machinery is largely absent, and much of the mechanism is borrowed from non-skeletal tissue. Metabolomic, epigenomic and skeletal-phenotype data are not yet integrated across layers, so causal chains are still assembled from correlation. Resolving the spatiotemporal heterogeneity of the remodeling surface will demand single-cell and spatial metabolomics. Finally, machine-learning integration of these multi-omic layers, though promising for target and response prediction, is constrained by annotated data and interpretability.

The merit of any framework is ultimately judged by the experiments it prescribes; we therefore close by enumerating the open questions in explicit form and in descending order of priority. Prerequisite to all else is the question of necessity—whether a macrophage metabolic node genuinely governs bone mass—a matter that can only be settled by assessing bone mass and microarchitecture in mice carrying myeloid- or osteoclast-lineage-restricted deletions of Hif1a, Cpt1a, Slc2a1 or Sirt1, phenotyped across ovariectomy, ageing, diabetes and diet-induced obesity, with node-selective rescue; no such dataset has yet been produced, and in its absence the axis cannot be adjudged causal. Next, and arguably the most tractable question raised here, is whether histone lactylation is present and dynamically regulated in bone-resident macrophages and osteoclast precursors, an issue demanding quantification of pan-lysine and H3K18 lactylation in sorted osteomacs and osteoclast precursors from healthy, ovariectomized and aged bone. Third, what substrates does the formation-supporting resident macrophage of bone actually consume, and does its program mirror a canonical M2 state or a bone-specific configuration that no polarization label currently captures? Fourth, single-cell and spatially resolved metabolomic and epigenomic profiling of the remodeling surface is needed to establish whether the metabolic states inferred from bulk populations persist in individual cells and how they are distributed relative to the bone surface, vasculature and marrow. Fifth, what share of the bone loss in ageing, diabetes and obesity is attributable to the macrophage, as opposed to estrogen withdrawal, direct glycation and matrix damage, or marrow adipocyte expansion? Sixth, do lactylation, succinylation and acetylation respond divergently to an inflammatory metabolic shift within the same bone macrophage population, as their distinct metabolic drivers would predict? Seventh, given the opposing roles of HIF-1α and mTOR in the macrophage versus the osteoblast–vascular compartment, can bone-restricted, macrophage-selective delivery yield a net anabolic effect, and what degree of selectivity suffices? Eighth, can vesicle-based, mitochondrial-transfer or gut–bone strategies be sustained safely over the multi-year horizon that osteoporosis therapy requires, and what immunological toll does chronic macrophage reprogramming exact? Lastly, are polarization or lactylation signatures detectable in accessible human samples, and do they predict bone loss or treatment response prospectively? Of these, settling the first and second would contribute most to determining whether metabolic–polarization coupling emerges as a genuine therapeutic axis in osteoporosis or remains an elegant account of correlated phenomena.

Progressively meeting these challenges, above all *in vivo* causal validation and cross-layer data integration, would advance the coupling from a concept into a stratifiable, predictable and druggable strategy. Each translational step would in turn test, refine and consolidate the metabolism–polarization coupling itself, closing the loop between framework, application and mechanistic proof.
